# Bacteriocins and the assembly of natural *Pseudomonas fluorescens* populations

**DOI:** 10.1111/jeb.13010

**Published:** 2016-12-21

**Authors:** J. B. Bruce, S. A. West, A. S. Griffin

**Affiliations:** ^1^ Department of Zoology University of Oxford Oxford UK

**Keywords:** bacteriocins, competition, *Pseudomonas fluorescens*, resource competition, soil

## Abstract

When competing for space and resources, bacteria produce toxins known as bacteriocins to gain an advantage over competitors. Recent studies in the laboratory have confirmed theoretical predictions that bacteriocin production can determine coexistence, by eradicating sensitive competitors or driving the emergence of resistant genotypes. However, there is currently limited evidence that bacteriocin‐mediated competition influences the coexistence and distribution of genotypes in natural environments, and what factors drive interactions towards inhibition remain unclear. Using natural soil populations of *Pseudomonas fluorescens*, we assessed the ability of the isolates to inhibit one another with respect to spatial proximity in the field, genetic similarity and niche overlap. The majority of isolates were found to produce bacteriocins; however, widespread resistance between coexisting isolates meant relatively few interactions resulted in inhibition. When inhibition did occur, it occurred more frequently between ecologically similar isolates. However, in contrast with results from other natural populations, we found no relationship between the frequency of inhibition and the genetic similarity of competitors. Our results suggest that bacteriocin production plays an important role in mediating competition over resources in natural settings and, by selecting for isolates resistant to local bacteriocin production, can influence the assembly of natural populations of *P. fluorescens*.

## Introduction

The majority of bacteria produce small antimicrobial toxins, known as bacteriocins, which kill or inhibit the growth of sensitive competitors (Riley & Wertz, 2002; Cascales *et al*., 2007). Bacteria avoid the harmful effects of these toxins by carrying an immunity gene or lacking appropriate cell surface receptors, allowing harm to be directed towards competitors (Ghequire & De Mot, 2014). The harm caused by bacteriocin production is often important for the successful invasion, or defence, of an established bacterial population: bacteriocin producers frequently invade and outcompete sensitive competitors in laboratory microcosm experiments (Riley & Gordon, 1999; Greig & Travisano, 2008; Wloch‐Salamon *et al*., 2008; Waite & Curtis, 2009; Libberton *et al*., 2015). However, despite widespread recognition of the importance of bacteriocins in mediating competition between genotypes, we still have a limited understanding of this process in natural environments (Hibbing *et al*. 2010; Whitaker, 2009). In particular, it remains unclear why bacterial genotypes inhibit some competitors and not others, and whether bacteriocin‐mediated interactions can influence the distribution of genotypes in free‐living bacterial populations.

The abundance of bacteriocin producing genotypes in nature is often assumed to be a consequence of competition for limited resources (Gordon *et al*., 2007). Inhibitory interactions are expected to occur more frequently between ecologically similar genotypes, which require more of the same resources, and less frequently between ecologically dissimilar competitors (Freilich *et al*., 2011). However, very closely related isolates, despite requiring similar resources, are often unable to inhibit each other due to shared resistance mechanisms (Hawlena *et al*., 2010a,b). Conflating predictions concerning ecological and genetic similarity suggests that inhibitory interactions should occur more frequently between genetically ‘intermediate’ competitors (Schoustra *et al*., 2012). Although inhibitory interactions do appear to occur more frequently between genetically ‘intermediate’ competitors in marine and host‐associated bacterial populations, direct evidence that competition over resources promotes inhibitory interactions remains scant (Cordero *et al*., 2012; Hawlena *et al*. 2012; Kinkel *et al*., 2014). Our limited understanding of bacteriocin‐mediated competition in natural settings also extends to the consequences of these behaviours. In the laboratory, bacteriocin production can influence the diversity of microcosm populations by eradicating sensitive competitors, or driving the emergence of resistant genotypes (Kerr *et al*., 2002; Le Gac & Doebeli, 2009; Abrudan *et al*., 2012). However, it remains unclear whether bacteriocin‐mediated interactions are strong enough, or occur frequently enough, to impact the distribution of genotypes in natural bacterial populations (Perez‐Gutierrez *et al*., 2013; Ghoul *et al*., 2015).

Here, we examine bacteriocin‐mediated interactions between natural isolates to reveal what factors promote inhibitory interactions in nature, and explore their consequences for the distribution of genotypes in soil. We used natural populations of the *Pseudomonas fluorescens* group, an important plant commensal, as our study system. We isolated *P. fluorescens* from soil at multiple sites in a local park and assessed the ability of isolates to inhibit each other with respect to spatial proximity. We expect that if bacteriocin production has influenced the distribution of genotypes in the population, inhibition would occur less frequently between coexisting isolates. We also constructed nutrient use profiles for each isolate, and measured genetic distances between isolate pairs, to examine interactions in the context of both the extent to which isolates are likely to compete for resources and genetic similarity.

## Material and methods

### Site description and sampling

We carried out sampling at multiple sites in University Parks, a managed parkland in Oxford consisting of sports fields, tended gardens and undisturbed parkland. We collected soil samples from eight sites in undisturbed regions of the park; the average distance between sites was approximately 250 m. At each site, we sampled a 1‐m transect consisting of four patches: a focal patch, and patches 1, 10 and 100 cm from the focal patch (Fig. S1). At each patch, we cleared surface debris and collected 1 cm^3^ of soil from the surface. We carried out all sampling in a single day in June 2014 and soil samples were stored in the laboratory at 23 °C and processed within 2 h.

### Sample processing and isolate identification

We isolated bacterial cells from soil samples as follows – 1 g of sample was suspended in 6 mL of minimal salts media (M9: 6.8 g Na_2_HPO_4_, 3 g KH_2_PO_4_, 0.5 g NaCl and 10 g NH_4_Cl (Sigma Aldrich Company Ltd., Gillingham, UK), per litre of dH_2_O) in 15‐mL falcon tubes. We vortexed soil suspensions vigorously and plated 80 μL of each suspension onto *Pseudomonas* isolation agar plates (47.4 g *Pseudomonas* isolation agar and 20 g glycerol per litre dH_2_O). Plates were incubated at 23 °C for 48 h before we randomly selected 30 isolates from each patch, transferred them to fresh KB agar plates (20 g protease peptone N^o^3 (Becton Dickinson U.K. Ltd., Oxford, UK) 10 mL glycerol, 1.5 g K_2_HPO_4_.3H_2_O, 12 g agar and 1.5 g MgSO_4_.7H_2_O (Sigma Aldrich Company Ltd), per litre of dH_2_O) and incubated overnight at 23 °C.

We used colony PCR to identify isolates belonging to species of the *P. fluorescens* group. PCRs contained 12.5 μL of 2× MangoMix (Bioline Reagents Ltd., London, UK), 10.5 μL of ddH_2_O, 0.5 μm of each primer (Table S1) and 1 μL of DNA template (colony pick suspended in 100 μL dH_2_O) up to a volume of 25 μL. The cycling conditions consisted of 5 min at 95 °C, 34 cycles of 94 °C for 30 s, 55 °C for 10 s, 72 °C for 45 s and a final extension of 72 °C for 5 min before cooling to 4 °C. All reactions were carried out with a positive (*P. fluorescens* SBW25), negative (*Pseudomonas aeruginosa* PAOl) and blank control. We established the presence or absence of amplified PCR product using gel electrophoresis.

We randomly selected seven positive isolates from each patch, in each site (seven isolates × four patches × eight sites = 224 isolates) for use in the study. These isolates were grown for 24 h at 23 °C on an orbital shaker at 200 rpm in 6 mL of KB media ((20 g protease peptone N^o^3 (Becton Dickinson U.K. Ltd), 10 mL glycerol, 1.5 g K_2_HPO_4_.3H_2_O and 1.5 g MgSO_4_.7H_2_O (Sigma Aldrich Company Ltd) per litre of dH_2_O) and frozen in 50% glycerol at −80 °C.

### Assaying inhibitory phenotypes of isolates

We assayed the ability of isolates from the focal patch to inhibit the growth of isolates from patches 1, 10 and 100 cm away in the same site. We also assessed their ability to inhibit isolates from the focal patch of other sites, allowing us to determine whether the frequency of inhibition caused by isolates increased with spatial distance from the focal patch locally (up to 1 m away) and further afield (between sites). We determined the ability of isolates to inhibit each other's growth with pyocins by testing whether an isolate supernatant inhibits the growth of other isolates. We spotted KB agar plates with supernatants, spread a lawn of each culture over the plate and recorded which supernatants inhibited which bacterial lawns. Assays were carried out in duplicate.

#### Supernatant extraction

To extract pyocin‐containing supernatants, we cultured 56 isolates (seven isolates from the focal patch of each site) for 24 h in 6 mL of KB media (23 °C at 200 rpm). We measured the cell density of cultures at A600 (SpectraMax), standardized each culture to an OD of ~0.3 and diluted this 10‐fold in fresh KB media. Cultures were incubated for 12 h before we added 100 μL of 0.02 mg μL^−1^ mitomycin C (Sigma, UKltd), a strong inducer of bacteriocin production, and incubated for a further 4 h (Pentermann *et al*., 2014). We then centrifuged cultures at ~13000 *g* for 10 min, obtaining a clear, cell‐free supernatant by filter sterilizing with a 0.02‐μm filter and storing at −20 °C until required.

#### Inhibition assays

We spotted KB agar plates with 15 μL of supernatant and allowed the plates to dry at room temperature. We cultured all 224 isolates from freezer stocks for 24 h in 6 mL of KB media (23 °C at 200 rpm). Cell density was standardized to an OD of ~0.5 and diluted 10‐fold in M9 before 70 μL of culture was spread onto the supernatant‐spotted KB agar plates. We incubated plates at 23 °C for ~12 h, or until a uniform lawn of bacterial growth was visible, and checked the plates for zones of inhibition on and around the supernatant spots and recorded whether a lawn was inhibited by a particular supernatant. Inhibition was recorded as a binary response (1 for inhibition, 0 for no inhibition). Plates were checked regularly after 12 h as zones of inhibition caused by some supernatants could become overgrown. We recorded patterns of inhibition and resistance in a 7 × 28 matrix for each set of within‐site interactions and a 56 × 56 matrix for between‐site interactions.

Each supernatant was spotted on a KB plate to ensure it was cell‐free. As a control, each lawn was also spotted with KB media treated identically to supernatant cultures, to demonstrate that residual mitomycin C was not responsible for zones of inhibition. Bacteriophage might also be responsible for inhibition on bacterial lawns, which manifest as areas of slightly reduced growth with characteristic plaques. We observed no plaque formation around the zones of inhibition on our plates and no evidence of self‐inhibition, suggesting bacteriophage was not responsible.

### Characterizing inhibitory molecules

We repeated the inhibition assays that resulted in one isolate inhibiting another in order to determine what mediated inhibitory interactions. *Pseudomonads* can produce an array of inhibitory molecules that vary greatly in size: large phage tail‐like toxins known as tailocins, smaller proteineous toxins known as S‐type pyocins and other assorted small inhibitory molecules (Ghequire & De Mot, 2014). We repeated inhibition assays after passing pyocin‐containing supernatants through different sized filters and determined whether isolates were capable of killing using large (>100 kDa), medium (>3 kDa and <100 kDa) or small (<3 kDa) molecules.

We obtained cell‐free supernatant from 35 isolates (those capable of inhibiting another isolate) as previously. To obtain supernatant containing large inhibitory molecules, we passed 500 μL of the cell‐free supernatant of each isolate through a 100‐kDa spin filter (EMD Millipore, Billerica, MA, USA). The flow through was discarded and the remaining pyocin‐containing supernatant was made up to 500 μL using M9 solution. To obtain supernatant containing small inhibitory molecules, we passed 500 μL of the cell‐free supernatant of each isolate through a 3‐kDa spin filter and retained the flow through. The flow through contains only particles smaller than 3 kDa; thus, all larger, potentially inhibitory molecules are removed from the supernatant. To obtain supernatant containing intermediately sized inhibitory molecules, we passed 500 μL of the cell‐free supernatant of each isolate through a 100‐kDa spin filter, and the flow through was collected and passed through a 3‐kDa filter. This flow through was discarded and the retained supernatant was made up to 500 μL using M9 solution. We then performed inhibition assays as previously and recorded which size fraction of their supernatants isolates were capable of inhibiting with.

### MLST of focal isolates

We used multilocus sequence typing (MLST) of four housekeeping genes to provide a measure of genetic similarity between isolate pairs, allowing us to explore the relationship between inhibition and genetic similarity of our isolates.

#### DNA extraction and PCR

We extracted genomic DNA using the Wizard Genomic DNA purification kit (Promega, Switzerland) and amplified four housekeeping genes (gyrB, RecA, RpoB and RpoD; Table S1.) for each of the focal isolates. All reactions were performed in 50 μL volume containing 1 U of DreamTaq polymerase (Thermo Scientific), 5 μL of DreamTaq Buffer, 100 mm of each primer, 0.2 mm of each dNTP, 40.75 μL of ddH_2_O and 20 ng of DNA template. We used the following cycling conditions: 95 °C for 3 min, then 35 cycles of 95 °C for 30 s, 58 °C/60 °C/64.5 °C for 30 s, 72 °C for 45 s and a final extension of 72 °C for 5 min. The annealing temperatures for each primer set are found in the Table S2.

#### Sequencing and analysis

Purified products were sent to SourceBioscience (Nottingham, UK) for Sanger sequencing with the respective primer pairs used for PCR amplification used as forward and reverse sequencing primers (Table S1). We checked the quality of the resulting sequences using Geneious Pro (Biomatters Ltd., Auckland, New Zealand), generating a consensus sequence for each isolate before trimming and aligning the sequences to obtain an identical length sequence for each gene in each isolate. We constructed a concatemer of all four genes for each isolate using Mega 6.0 software (Tamura *et al*., 2013). From this, we calculated pairwise genetic distances between isolate pairs using the Jukes‐Cantor model and constructed a neighbour‐joining tree showing the relationships between focal isolates.

### Resource competition

We assayed the ability of each isolate to grow on different carbon sources and calculated a measure of niche overlap between isolate pairs, allowing us to explore the relationship between inhibition and resource use in our isolates.

We measured the growth of each isolate on different carbon sources, in duplicate, using EcoPlates (Biolog, Inc., Hayward, CA, USA). EcoPlates plates contain 31 different carbon sources commonly found in soil. We cultured all 56 isolates for 24 h in 6 mL of KB media (23 °C at 200 rpm) before standardizing cultures to an OD of ~0.25 and diluting 10‐fold. We inoculated each well of the plates with 150 μL of culture and incubated at 23 °C for 48 h. We measured the absorbance at A_600_ immediately after inoculation and after 48 h. The absorbance of the control well was subtracted from the reading for each well and a measure of niche overlap was calculated (Kinkel *et al*., 2014).

$$\begin{array}{cc} \text{Niche overlap} & =\{(\sum \text{minimum absorbance}\;\;\;\;a,b)/\text{total absorbance}\;\;a\\ & +\left(\sum \text{minimum absorbance}\;\;\;a,b)/\text{total absorbance}\;\;\;b\right)\}/2 \end{array}$$

where (minimum absorbance *a*,* b*) is the minimum absorbance value for a pair of isolates (*a* and *b*) on a given nutrient; these values are summarized over all 31 nutrients. The total absorbance for an isolate is the sum of absorbance values for that isolate over all 31 nutrients. Therefore, niche overlap between a pair of isolates is the mean proportion of total nutrient use that overlaps between the two isolates.

### Statistical analysis

We tested for significant differences in inhibition caused by isolates within their site and inhibition they caused between sites using a generalized linear mixed model (GLMM). We used inhibition between isolates as a binary response variable and distance (within site or between site) as the explanatory variable and included producing isolate and lawn isolate as random effects to account for the fact isolates are used in multiple comparisons. We also tested for significant differences in inhibition caused by focal isolates against isolates from other patches within sites using GLMM, with inhibition as a binary response variable and spatial distance between isolates as the explanatory variable. We included site and isolate as random effects to take into account the multiple sites used in the analysis and that isolates were used in multiple comparisons, respectively.

We wish to determine whether genetic similarity between isolates could explain whether isolates were more or less likely to inhibit another isolate. To determine whether there was any correlation between frequency of inhibition and genetic distance between isolates, we used a generalized linear model (GLM) with inhibition as a binomial response variable and genetic distance between isolates as the explanatory variable.

Finally, to determine whether there was any correlation between frequency of inhibition and niche overlap, we used a GLM with inhibition as a binomial response variable and niche overlap between isolates as the explanatory variable.

## Results

### Isolate collection and inhibition profiles

We selected seven isolates from a single patch at each of our eight sites (56 isolates) for further analysis, the majority of which were genetically distinct. MLST analysis of four housekeeping genes revealed that 39 of the 56 focal isolates were unique (70%) and the average pairwise genetic distance between isolates was 0.031, ranging from 0.000 to 0.047. A maximum‐likelihood (ML) tree of our focal isolates with type strains of the *P. fluorescens* complex suggests that our isolates are part of the *P. fluorescens* group within the complex and that they are comprised of multiple different species (Fig. S2). Rarefaction analyses suggest that a considerable portion of diversity present in the population was sampled (Fig. S3).

**Figure 1:**
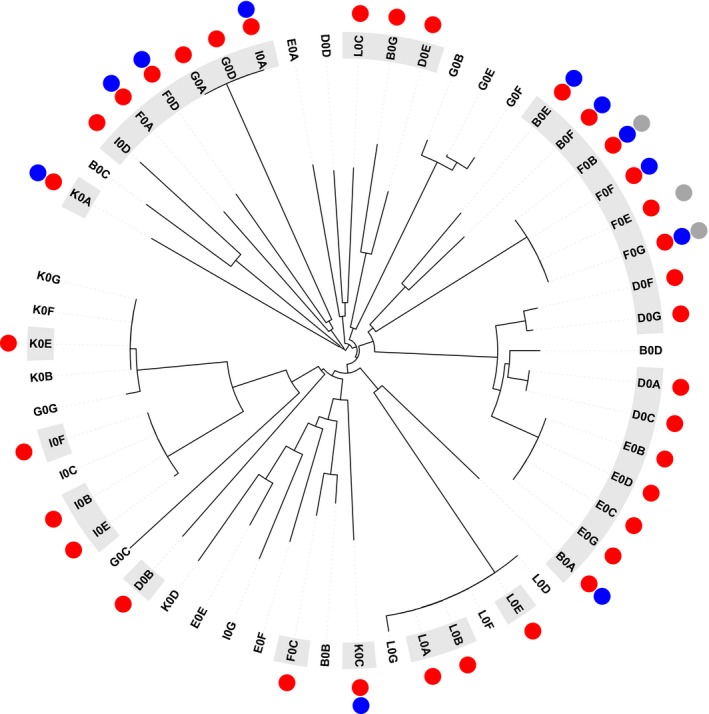
Inhibitory capacity and phylogeny of isolates based on four housekeeping genes. Outer ring highlights inhibitory isolates (grey) and noninhibitory isolates (white). Coloured circles indicate the ability to inhibit another isolate with large particles (>100 kDa, red), medium particles (<100 kDa, blue) and small particles (<3 kDa, grey). The majority of isolates are genetically distinct and are capable of producing at least one type of inhibitory molecule.

**Figure 2:**
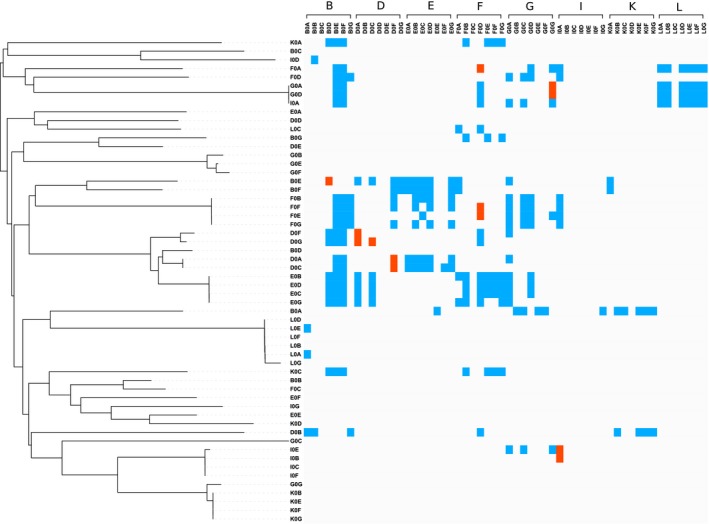
Phylogeny and inhibition profiles of the isolates. The left‐hand side shows a phylogenetic tree of all isolates, whereas the top shows isolates grouped by sampling site. Grey squares indicate no inhibition, blue squares indicate an inhibitory interaction between isolates from different sites (between sites) and red squares indicate inhibitory interactions between isolates from the same site (within sites). The majority of interactions do not result in inhibition, whereas genetically identical isolates can have different inhibition and resistance profiles.

The majority of isolates produced a molecule capable of causing inhibition, and were inhibited by at least one of the bacteriocins produced by isolates in our collection. Approximately two‐thirds (63%) of isolates produced an inhibitory molecule that could inhibit the growth of at least one other isolate and over three quarters (79%) of isolates were inhibited by at least one other isolate (Fig. 1). In only 7% of interactions did the supernatant of one isolate inhibit the growth of another isolate (Fig. 2).

The inhibitory molecules produced by the isolates in our collection varied in size, and, therefore, likely represent a range of different bacteriocin types. We found that isolates produce large (e.g. R and F types), intermediate (e.g. S types) and small (unknown) inhibitory molecules. All inhibitory isolates can inhibit using products >100 kDa in size, whereas 20% and 9% of inhibitory isolates were capable of inhibiting with medium (<70 kDa) and small (<3 kDa) products, respectively. We find that isolates with 100% sequence similarity at four housekeeping genes can show different patterns of inhibition and resistance (Fig. 2).

### Inhibition, distance, genetic distance and niche overlap

An analysis incorporating all the factors used in the study suggests significant differences in inhibition attributable to the distance between isolates and the degree of niche overlap, but not the genetic distance between isolates (Table S3).

**Figure 3:**
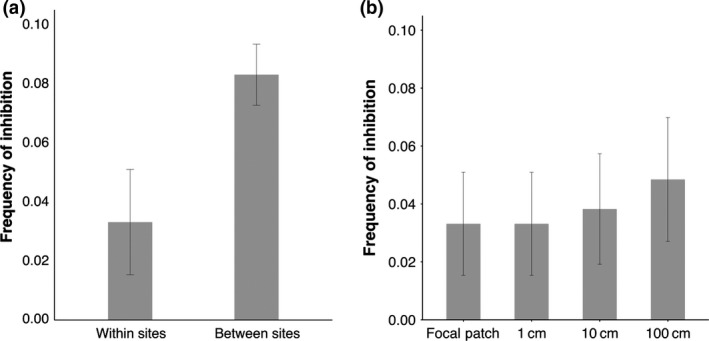
Frequency of inhibitory interactions between isolates. (a) The frequency of inhibitory interactions between coexisting isolates (within sites, *n* = 372) is significantly lower than that between non‐coexisting isolates (between sites, *n* = 2744). (b) The frequency of inhibitory interactions observed between coexisting isolates and in interactions between isolates separated by 1, 10 and 100 cm within sites (*n* = 392 for each distance). Error bars indicate 95% confidence intervals.

We found that inhibition occurred less frequently between isolates from the same site, and more frequently between isolates from different sites (Fig. 3a; GLMM: *Z* = 4.796, *P* = 1.62 × 10^−6^). There was also a trend, within sites, for inhibition to occur more frequently between isolates separated by greater distances, but this relationship was not significant (Fig. 3b; GLMM: *Z* = 1.130, *P* = 0.259).

**Figure 4:**
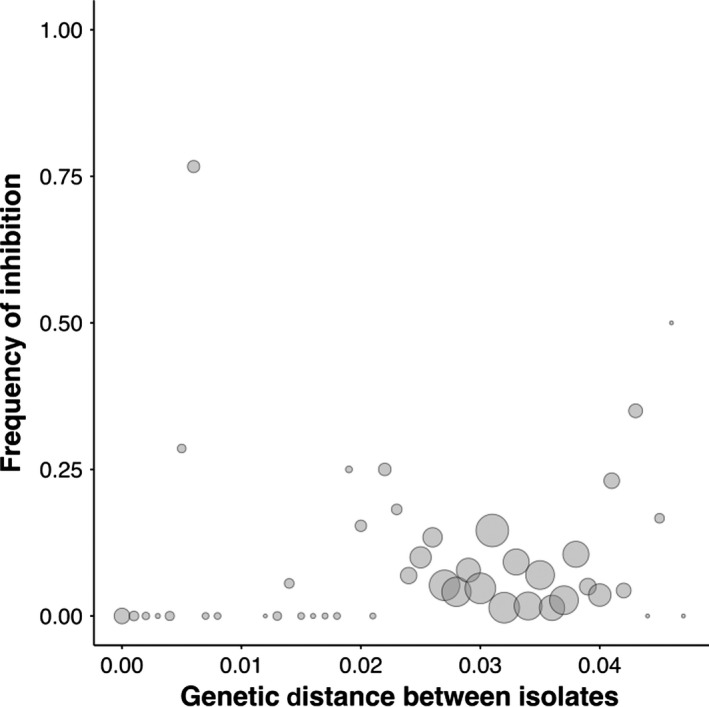
Inhibitory interactions and genetic distance between isolates. Inhibitory interactions occur no more frequently between genetically similar isolates than between genetically dissimilar isolates. The size of the point reflects the number of interactions: smallest points indicate <5 interactions and largest points indicate >200 interactions (*n* = 3080).

**Figure 5:**
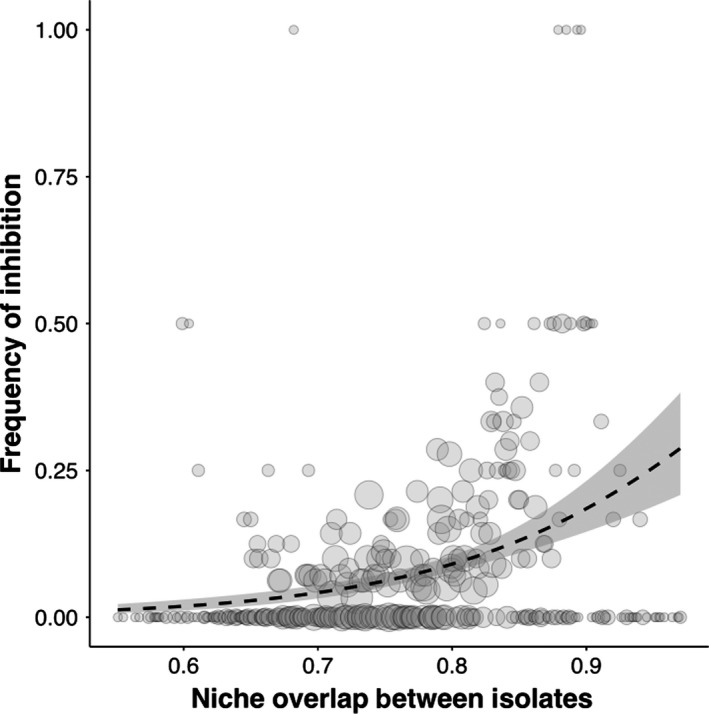
Inhibitory interactions and niche overlap between isolates. Inhibitory interactions occur more frequently between isolates with greater niche overlap. The size of the points reflects the number of interactions, smallest points indicate <2 interactions and largest points indicate >24 interactions (*n* = 3080).

We find that isolates were no more likely to inhibit genetically similar isolates than any other isolate (Fig. 4; GLM: *t*_44_ = −0.89, *P* = 0.37) but that inhibition occurred more frequently between isolates with greater niche overlap (Fig. 5; GLM: *t*_329_ = 7.02, *P* = 1.23e^−11^).

## Discussion

Our results show that natural populations of *P. fluorescens* are intensely competitive: the majority of isolates produce bacteriocins and some are capable of producing two or more types (Fig. 1). However, resistance to bacteriocins is so widespread that relatively few interactions actually result in one isolate inhibiting another (Fig. 2). In contrast with previous studies, we find no evidence that inhibition occurs more frequently between genetically more similar or more dissimilar isolates (Fig. 4). However, we do show that inhibition occurs more frequently when competitors have greater niche overlap, suggesting bacteriocins are important in mediating competition over resources in natural settings (Fig. 5). Finally, we found that inhibitory interactions occur significantly less frequently between coexisting isolates, suggesting that bacteriocin production plays a role in shaping natural *P. fluorescens* populations by selecting for isolates resistant to local bacteriocin producers (Fig. 3).

Despite the potential for bacteriocin‐mediated conflict, resistance appears to be so widespread that relatively few interactions actually result in one isolate inhibiting another. Resistance to the bacteriocins of conspecifics is common in other bacterial populations: in natural *Vibrio* and *Bacillus* populations, <10% of interactions resulted in inhibition despite approximately half of isolates producing bacteriocins (Cordero *et al*., 2012; Perez‐Gutierrez *et al*., 2013). Our results provide further evidence that resistance is prevalent and either easily acquired, or easily transferred between isolates, in natural settings. Acquiring resistance is thought to come at a significant cost to the cell, yet resistance was the most common outcome of competition even between isolates unlikely to encounter each other frequently (Opsata *et al*., 2010; Inglis *et al*., 2016). Why maintain costly resistance against the toxins of rarely encountered competitors? Bacteriocins, such as the phage tail‐like toxins produced by our isolates, often target essential components of the cell membrane, such as siderophore or lipopolysaccharide (LPS) receptors (Baysse *et al*., 1999; Cascales *et al*., 2007; Kohler *et al*., 2010; Elfarash *et al*., 2012) . Mutations in these receptors can confer resistance to bacteriocins, and a single mutation may, by altering the structure of surface receptors, confer resistance against multiple bacteriocins (Scanlan *et al*., 2015).

We found that bacteriocin‐mediated inhibition occurs more frequently between competitors with similar resource requirements. Competition over limited resources is thought to be a key driver in the evolution of competitive phenotypes and selection for bacteriocin production is likely strongest when resource competition is intense (Cornforth & Foster, 2013). By producing bacteriocins that harm ecologically similar competitors, isolates maximize the fitness benefits of costly toxin production, as most resources liberated are beneficial to the producer (Schoustra *et al*., 2012). Our results suggest that resource competition is an important factor driving the evolution of bacteriocin production in natural populations and are consistent with the limited evidence available from other bacterial populations: inhibitory interactions between *Streptomyces* are often more intense when competitors have similar resource requirements (Kinkel *et al*., 2014; Schlatter & Kinkel, 2014). Directing harm towards stronger resource competitors also allows isolates to avoid engaging in costly conflict with ecologically distinct competitors, which may partly explain the coexistence of multiple *P. fluorescens* genotypes at each site in our study.

Contrary to expectations, we found no evidence that inhibition occurs more frequently between genetically more similar or dissimilar competitors. Resource competition is thought to be strongest between genetically similar competitors (Freilich *et al*., 2011; Jousset *et al*., 2011). Consequently, with the exception of competition between identical or very closely related isolates, inhibition is expected to occur more frequently between genetically similar competitors. However, our results emphasize that resource competition is the key factor driving interactions towards inhibition in natural populations. While harming strong resource competitors will often equate to more frequent inhibitory interactions between nonidentical, genetically similar competitors, as observed in a recent study of clinical and laboratory *P. aeruginosa* isolates*,* the relationship is likely weaker in natural settings where resource use can vary significantly between even closely related isolates (Belotte *et al*., 2003; Schoustra *et al*., 2012; Kraemer & Kassen, 2015). Our results suggest that although genetic similarity will often be a good predictor of the potential for inhibition between competitors, understanding what drives interactions towards inhibition in natural settings will also require knowledge of the resource requirements of each competitor.

Finally, we found that bacteriocin‐mediated inhibition occurs less frequently between coexisting isolates, suggesting bacteriocin production selects for genotypes resistant to local bacteriocin producers. The low levels of inhibition we observe between coexisting isolates could occur if (i) isolates interact more frequently with identical or closely related competitors, who produce similar bacteriocins and are thus resistant, or (ii) if unrelated isolates have evolved resistance to local bacteriocin producers (Kerr *et al*., 2002; Ghoul *et al*., 2015). These outcomes are not mutually exclusive: each of our sites is genotypically diverse, whereas many harbour a numerically dominant genotype, suggesting both processes may be important. Attenuation of inhibitory interactions between coexisting isolates is also reported in *Bacillus* populations found in marine sediment, which raises the question of why bacteriocin production is maintained in these populations when resistance is prevalent locally (Perez‐Gutierrez *et al*., 2013). One explanation is that bacteriocin production provides a fitness advantage in competition with invading genotypes: recent empirical studies have shown that invasion of a population can be prevented by bacteriocin production and that genotypically rich populations are often less invasible than their homogenous counterparts (Jousset *et al*., 2011; Libberton *et al*., 2015). The genotypically diverse, locally resistant patches of bacteriocin producers we find at each of our sites may represent structured, stable collections of genotypes resistant to invasion by conspecific competitors.

Overall, our results suggest an important role for bacteriocin production in natural free‐living bacterial populations. We provide empirical evidence that suggests bacteriocin production mediates competition over resources in natural bacterial populations, and show that bacteriocin production can influence the assembly of bacterial populations by selecting for isolates resistant to local bacteriocins.

## Supporting information


**Data S1** Supplemental Material and Methods.
**Figure S1** Transect and sampling sites.
**Figure S2** Phylogenetic tree of focal isolates and *Pseudomonas fluorescens* complex type strains.
**Figure S3** Rarefaction curve of the focal isolates in this study.
**Table S1** Primer sequences.
**Table S2** PCR and sequencing primers and annealing temperatures.
**Table S3** Analysis incorporating distance, niche overlap and genetic distance.
