# Supplementary material for: Bacteriocins and the assembly of natural Pseudomonas fluorescens populations
Source: J Evol Biol. 2016 Dec 21;30(2):352–60. doi: 10.1111/jeb.13010 (PMC6849615; doi:10.1111/jeb.13010)
Supplement: Supplementary file 1 — Data S1 Supplemental Material and Methods. Figure S1 Transect and sampling sites. Figure S2 Phylogenetic tree of focal isolates and Pseudomonas fluorescens complex type strains. Figure S3 Rarefaction curve of the focal isolates in this study. Table S1 Primer sequences. Table S2 PCR and sequencing primers and annealing temperatures. Table S3 Analysis incorporating distance, niche overlap and genetic distance. [file JEB-30-352-s001.docx]

**Supplementary Information**

*Supplemental Material and Methods*

**Phylogeny**

The Multi-Locus Sequence Analysis (MLSA) phylogeny was constructed using our 56 focal isolates and type strains representing each group of the *P.fluorescens* complex. Type strains are described in Andreoni (2014) and Garrido-Sanz (2016) and partial sequences for the gyrB, recA, rpoB and rpoD housekeeping genes for type strains were retrieved from GenBank (Bensen 2011). Sequences were aligned using MUSCLE (Edgar 2004), alignments cut and concatenated using MEGA software (Tamura 2013). To obtain a phylogeny we used the Maximum-Likelihood (ML) method and the Tamura-Nei model with 10000 bootstraps.

**Rarefaction curves**

From a concatenation of 4 housekeeping genes (gyrB, recA, rpoB and rpoD) in our focal isolates we produced a presence/absence matrix of polymorphisms at all variable sites in the alignment. We scored each polymorphism, rather than every polymorphic site, as many sites segregated for 3 or 4 nucleotides. The matrix was used as the input to the program EstimateS 8.2 (Colwell 2013) to create rarefaction curves. For the analysis, we treated individual sequences as samples and individual polymorphisms as ‘species’ after Vos et al (Vos 2008).

**Statistical analysis**

We carried out an analysis incorporating the multiple factors used in our study (Distance, Niche Overlap and Genetic Distance). We used a generalized linear mixed effects model (GLMM) with inhibition between isolates as a binary response variable; distance, niche overlap and genetic distance between isolates as explanatory variables and included producing isolate and lawn isolate as random effects to account for the fact isolates are used in multiple comparisons.

*
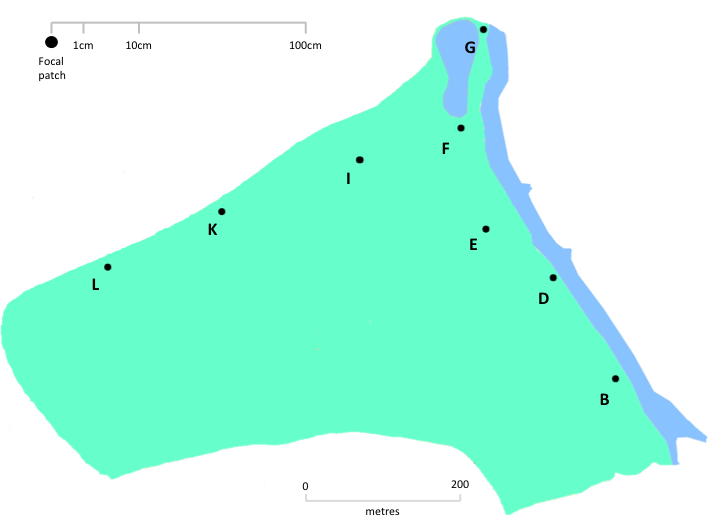
Supplementary Figures*

**Figure S1 – Transect and sampling sites.** A schematic map of University Parks, Oxford, showing sampling sites used in the study and a schematic of the transect sampled at each site.

**
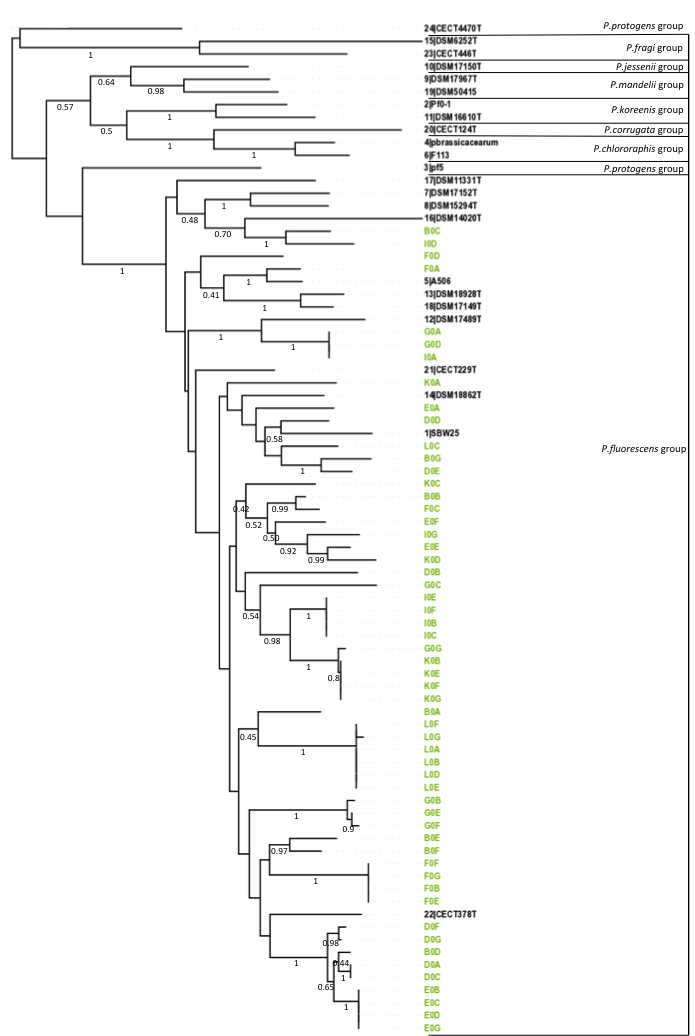
Figure S2 – Phylogenetic tree of focal isolates and *P.fluorescens* complex type strains.** A phylogenetic tree of 56 focal isolates and type strains from each group of the *P.fluorescens* complex based on partial sequences of the gyrB, recA, rpoB and rpoD genes and constructed using the ML method and the Tamura-Nei model. Only bootstrap values above 40% (from 10000 replicates) are shown. Black text indicates type strains and green text indicates isolates from this study.

**
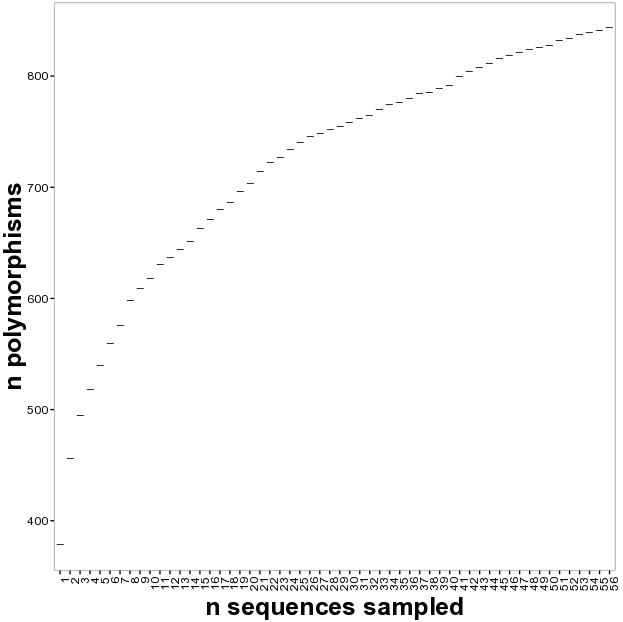
**

**Figure S3 – Rarefaction curve of the focal isolates in this study.** The number of polymorphisms in a concatemer of 4 housekeeping genes (gyrB, recA, rpoB and rpoD) as a function of sample size.

*Supplementary tables*

| **Table S1. Primer Sequences** | |  |  |
| --- | --- | --- | --- |
|  |  |  |  |
| **Primer** | **Gene Product** | **Sequence** | **Length** |
| 16S_F | 16S ribosomal RNA | AGAGTTTGATCCTGGCTCAG | 922bp |
| 16S_R |  | CTACGGCTACCTTGTTACGA |  |
| gyrB_F | DNA gyrase subunit B | GGTGGTCGATAACTCCATCG | 492bp |
| gyrB_R |  | CGCTGAGGAATGTTGTTGGT |  |
| recA_F | recombinase A | TGGCTGCGGCCCTGGGTCAGATC | 435bp |
| recA_R |  | ACCAGGCAGTTGGCGTTCTTGAT |  |
| rpoB_F | RNA polymerase subunit beta | TGGCCGGTCGTCACGGTAACA | 477bp |
| rpoB_R |  | CCGAAACGCTGACCACCGAAC |  |
| rpoD_F | RNA polymerase sigma factor | CTGATCCAGGAAGGCAACATCGG | 480bp |
| rpoD_R |  | ACTCGTCGAGGAAGGAGCG |  |

| **Table S2. PCR and Sequencing Primers and Annealing temperatures** | | |  |  |
| --- | --- | --- | --- | --- |
|  |  |  |  |  |
| **Gene** | **Forward** | **ºC** | **Reverse** | **ºC** |
| 16S | 16S_F | 58 | 16S_R | 58 |
| gyrB | gyrB_F | 58 | gyrB_R | 58 |
| recA | recA_F | 60 | recA_R | 60 |
| rpoB | rpoB_F | 64 | rpoB_R | 64 |
| rpoD | rpoD_F | 58 | rpoD_R | 58 |

**Table S3. Analysis incorporating Distance, Niche Overlap and Genetic Distance**

|  | |  | |  | |  | |  | |
| --- | --- | --- | --- | --- | --- | --- | --- | --- | --- |
| **Formula:** | Inhibition ~ Distance + Niche Overlap + Genetic Distance + (1\|Producer Isolate) + (1\|Victim Isolate) | | | | | | | |  |
| **Family:** | Binomial | |  | |  | |  | |  |
|  | |  | |  | |  | |  | |
| *Fixed Effects:* | |  | |  | |  | |  | |
|  | | **Estimate** | | **Std.Error** | | **Z Value** | | **Pr(>\|z\|)** | |
| ***Intercept*** | | -11.628 | | 1.6179 | | -7.187 | | 6.62E-13 | |
| **Distance** | | 2.0447 | | 0.4039 | | 5.063 | | 4.14E-07 | |
| **Niche Overlap** | | 7.6231 | | 1.7065 | | 4.467 | | 7.93E-06 | |
| **Genetic Distance** | | -23.7563 | | 12.8062 | | -1.855 | | 0.0636 | |
|  | |  | |  | |  | |  | |
|  | |  | |  | |  | |  | |
| *Random Effects:* | |  | |  | |  | |  | |
|  | |  | |  | |  | |  | |
|  | | **Name** | | **Variance** | | **Std. Dev** | |  | |
| **Producer Isolate** | | Intercept | | 5.751 | | 2.398 | |  | |
| **Victim Isolate** | | Intercept | | 1.573 | | 1.254 | |  | |

*Literature Cited*

Andreani, N.A., Martino, M.E., Fasolato, L., Carraro, L., Montemurro, F., Mioni, R., Bordin, P. and Cardazzo, B. 2014. Tracking the blue: A MLST approach to characterize the *Pseudomonas fluorescens* group. *Food Microbiol.* **39**: 116-126

Bensen, D.A., Karsch-Mizrachi, I., Clark, K., Lipman, D.J., Ostell, J. and Sayers, E.W. 2011. GenBank. *Nucleic Acids Res* **39:** 32 – 37

Colwell, R.K. 2013. EstimateS: Statistical estimation of species richness and shared species from samples. Version 9 and earlier. User’s guide and application. <http://purl.oclc.org/estimates.>

Edgar, R. C. 2004. MUSCLE: multiple sequence alignment with high accuracy and high throughput. *Nucleic Acids Res* **32**:1792-1797.

Tamura, K., Stecher, G., Peterson, D., Filipski, A. and Kumar, S. 2013 MEGA6: Molecular Evolutionary Genetics Analysis Version 6.0. *Mol. Biol. Evol*. **30**: 2725-2729.

Garrido-Sanz, D., Meier-Kolthoff, J.P., Goker, M., Martin, M., Rivilla, R. and Redondo-Neito, M. 2016. Genomic and Genetic Diversity within the *Pseudomonas fluorescens* Complex. *PLoS One*. 11: 2

Vos, M. and Velicer, G.J. 2008. Isolation by Distance in the Spore-Forming Soil Bacterium *Myxococcus xanthus*. *Curr. Biol.* **18**: 286 – 391.
